# Case report: Suspected adrenal crisis after non-functional adrenal tumor resection: a case of temporal association with perioperative dexamethasone

**DOI:** 10.3389/fendo.2026.1829308

**Published:** 2026-07-21

**Authors:** Shuai He, Lifang Tan, Jing Yang, Chunxia Li, Yuchen Tan, Juan Shen, Cong Wang, Jun Zheng, Yongquan Wang

**Affiliations:** 1 Department of Urology, The First Affiliated Hospital (Southwest Hospital) of Army Medical University, Chongqing, China; 2 Institute of Urology, The First Affiliated Hospital (Southwest Hospital) of Army Medical University, Chongqing, China; 3 Department of Anaesthesiology, The First Affiliated Hospital (Southwest Hospital) of Army Medical University, Chongqing, China; 4 International Centre for Aging and Cancer, Hainan Medical University, Haikou, Hainan, China

**Keywords:** case report, dexamethasone, non-functional adrenal tumor, perioperative management, suspected adrenal crisis

## Abstract

Perioperative dexamethasone is commonly used in enhanced recovery protocols, yet its safety in adrenalectomy remains underevaluated. We report a 33-year-old woman who developed postoperative adrenal crisis–like deterioration 54 hours after right adrenalectomy for a radiologically and biochemically non-functioning adrenal tumor on basal preoperative testing. A single 5 mg intravenous dose of dexamethasone was administered intraoperatively for antiemetic prophylaxis. During clinical deterioration, the patient exhibited marked suppression of ACTH and cortisol, consistent with pharmacological glucocorticoid effects in the perioperative period. However, the clinical presentation was non-specific and alternative postoperative complications, including infection, thromboembolic disease, and metabolic disturbances, could not be fully excluded. The patient improved following combined supportive therapy and stress-dose hydrocortisone. Preoperative endocrine evaluation did not include a 1 mg overnight dexamethasone suppression test, and mild autonomous cortisol secretion could not be excluded. Dynamic assessment of adrenal reserve was not performed preoperatively. This case highlights that perioperative dexamethasone may be temporally associated with postoperative endocrine perturbations that mimic adrenal crisis in susceptible patients. However, causality cannot be established, and careful differential diagnosis is essential in the postoperative setting following adrenal surgery.

## Introduction

Adrenal incidentalomas, detected in approximately 5% of the general population, present considerable management challenges (1–3). Their increasing detection, driven by heightened health awareness and the widespread use of cross-sectional imaging, has led to more patients undergoing adrenalectomy (4–7). Current guidelines offer clear recommendations for the preoperative evaluation of cortisol excess, including mild autonomous cortisol secretion (MACS), yet provide limited guidance on the perioperative management of patients with biochemically non-functional tumors (8).

Compounding this ambiguity is the routine use of perioperative dexamethasone, a potent glucocorticoid with antiemetic and analgesic properties within enhanced recovery protocols (9, 10). Although a single dose is generally considered safe in patients with normal adrenal function (8), its prolonged biological activity may transiently suppress hypothalamic–pituitary–adrenal (HPA) axis function and complicate postoperative endocrine interpretation in selected clinical settings.

Here, we report a patient with a radiologically and biochemically non-functioning adrenal tumor on basal preoperative testing who developed postoperative adrenal crisis–like deterioration 54 hours after right adrenalectomy. A single intraoperative dose of dexamethasone was administered for antiemetic prophylaxis. This case highlights perioperative endocrine interpretation in adrenal surgery.

## Case presentation

**Figure 1:**
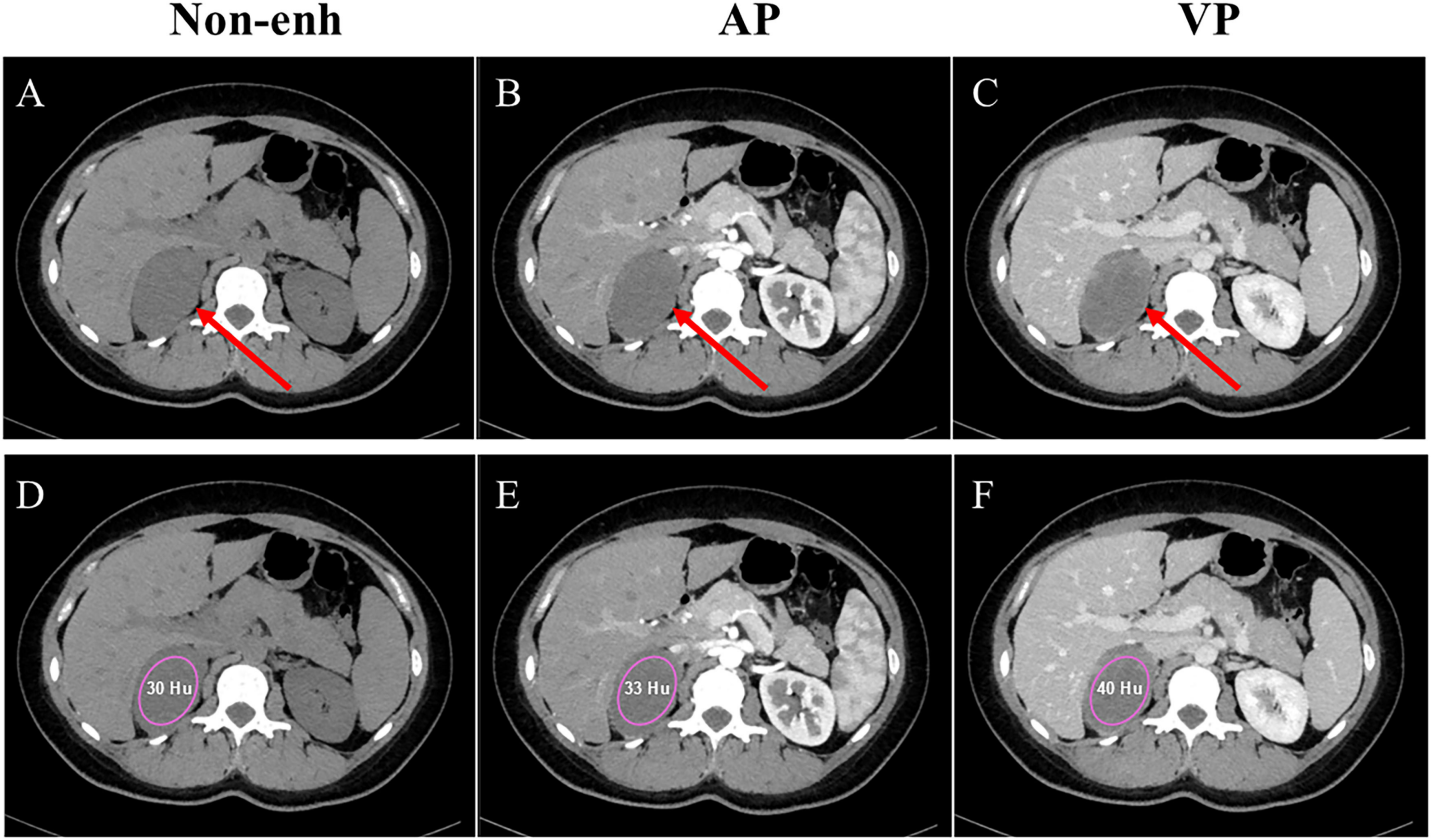
Preoperative abdominal CT of the right adrenal mass. Axial images demonstrate a well-circumscribed homogeneous right adrenal lesion (arrows) across unenhanced, arterial, and venous phases **(A–C)**. Region-of-interest attenuation values are 30, 33, and 40 Hounsfield units (HU), respectively **(D–F)**. Non-enh, AP, and VP indicate unenhanced, arterial phase, and venous phase imaging, respectively.

A 33-year-old asymptomatic woman was referred for evaluation of a right adrenal mass discovered incidentally on a postpartum ultrasound. Her medical history was notable only for a caesarean section two years prior, with no long-term medication use (including glucocorticoids), family history of endocrine or autoimmune disorders, smoking, or alcohol use. Follow-up CT demonstrated interval growth of the lesion from 5.5 × 3.9 cm to 6.7 × 4.4 cm. The patient was normotensive (120/79 mmHg; reference range, 90–140/60–90 mmHg) and normokalemic (serum potassium, 4.22 mmol/L; reference range, 3.5–5.0 mmol/L). Physical examination revealed no orthostatic hypotension, abdominal mass, cushingoid features, or clinical signs suggestive of adrenal insufficiency, primary aldosteronism, or pheochromocytoma. Adrenal CT revealed a well-circumscribed homogeneous right adrenal mass. Attenuation values were 30 HU on unenhanced imaging, 33 HU during arterial phase, and 40 HU during venous phase. Absolute and relative washout calculations were not performed (**Figure 1**, **Supplementary Figure 1**).

Basal morning hormonal evaluation showed no biochemical evidence of catecholamine, aldosterone, or overt cortisol excess. Morning serum cortisol was 27.06 μg/dL (reference range, 6.58–28.51 μg/dL), ACTH was 30.49 pg/mL (reference range, 5.00–60.00 pg/mL), aldosterone was 115.59 pg/mL (reference range, 65.00–295.70 pg/mL), renin was 5.02 ng/mL/h (reference range, 0.93–6.56 ng/mL/h), and the aldosterone-to-renin ratio was 2 (reference, <30). Plasma metanephrines and normetanephrines were also within normal limits (**Supplementary Table 1**). A 1 mg overnight dexamethasone suppression test (DST) was not performed as part of the preoperative workup; therefore, MACS could not be definitively excluded.

**Figure 2:**
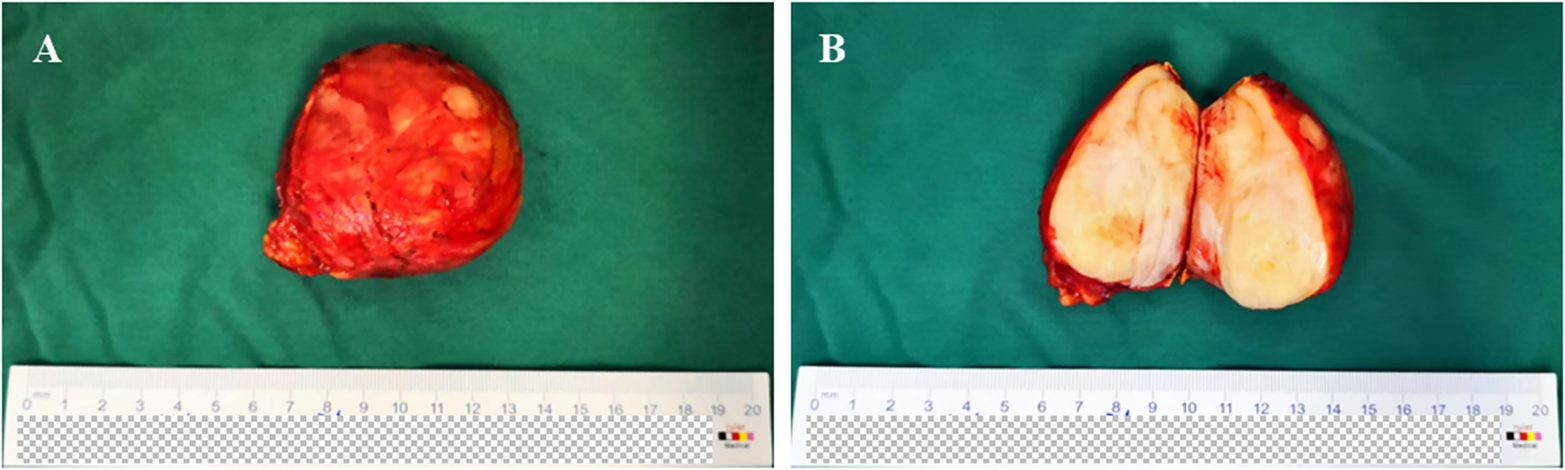
Gross pathological specimen of the resected adrenal tumor. **(A)** External appearance of the resected tumor. **(B)** Cut surface showing a solid, greyish-white, and firm mass with a whorled texture. A rim of residual adrenal tissue is visible at the periphery. The ruler is marked in centimeters.

In accordance with guidelines addressing tumor size (>4 cm) and unenhanced CT attenuation >20 Hounsfield units (8), the patient underwent retroperitoneal laparoscopic right adrenalectomy. During anesthetic induction, a single 5 mg intravenous dose of dexamethasone was administered for antiemetic prophylaxis. The resected specimen displayed a solid, greyish-white, firm cut surface with a whorled texture (**Figure 2**). Histopathological examination confirmed a ganglioneuroma composed of mature ganglion cells and Schwannian stroma. Immunohistochemistry was positive for S-100 and SOX10, with only isolated chromogranin A-positive ganglion cells; Ki-67 was <1%. SF-1 was negative, confirming the absence of adrenocortical differentiation. The adjacent adrenal cortex was histologically unremarkable, with no evidence of atrophy.

The early postoperative course was initially stable. At approximately 12 hours postoperatively, the patient developed mild hypotension (93/57 mmHg), which responded to fluid resuscitation without vasopressor support. Laboratory testing showed stable blood counts, electrolytes, and organ function (white blood cell count 9.60 × 10^9/L, neutrophils 8.36 × 10^9/L, hemoglobin 122 g/L, sodium 138.9 mmol/L, potassium 4.07 mmol/L, bicarbonate 22.7 mmol/L, and no evidence of major renal or hepatic dysfunction). At 31 hours, fever (38.5 °C) developed. At 50 hours, transient dyspnea occurred. Laboratory testing showed a white blood cell count of 7.59 × 10^9/L, neutrophil count of 5.82 × 10^9/L, hemoglobin of 112 g/L, and procalcitonin of 0.11 ng/mL. D-dimer was mildly elevated (1.13 mg/L FEU), while fibrin degradation products remained within the reference range.

At 54 hours postoperatively, the patient developed tachycardia (128 bpm), tachypnea (28/min), hypoxaemia (SpO_2_ 88% on room air), hyperpyrexia (39.5 °C), and cool clammy extremities, while remaining neurologically intact. Arterial blood gas analysis around this period showed pH 7.38, PaCO2–41 mmHg, PaO2–77 mmHg (FiO2 0.41), lactate 0.60 mmol/L, sodium 134 mmol/L, potassium 3.20 mmol/L, and bicarbonate 24.3 mmol/L. Endocrine testing at this time demonstrated suppressed endogenous cortisol (0.47 μg/dL) and ACTH (1.43 pg/mL). Repeat testing showed potassium 3.41 mmol/L with normal cardiac troponin I, CK-MB mass, and myoglobin, and a white blood cell count of 6.81 × 10^9/L. Blood and urine cultures, postoperative chest imaging, postoperative electrocardiography, lower-limb venous ultrasonography, and CT pulmonary angiography were not performed during the acute deterioration due to clinical urgency.

Management included intravenous glucose-saline infusion (approximately 1000 mL initially followed by 1500 mL), oxygen supplementation via face mask then nasal cannula, and antipyretic therapy. No vasopressor support was required. Stress-dose hydrocortisone was administered as a 100 mg intravenous bolus followed by continuous infusion at 200 mg/24 h. Clinical stabilization occurred after combined supportive management; therefore, the specific contribution of hydrocortisone cannot be isolated. Subsequent laboratory testing approximately 24 hours after initiation of hydrocortisone showed serum cortisol 22.77 μg/dL and ACTH 19.74 pg/mL. These values cannot be interpreted as evidence of endogenous HPA-axis recovery, as exogenous hydrocortisone is detected by cortisol immunoassays and suppresses ACTH secretion.

**Figure 3:**
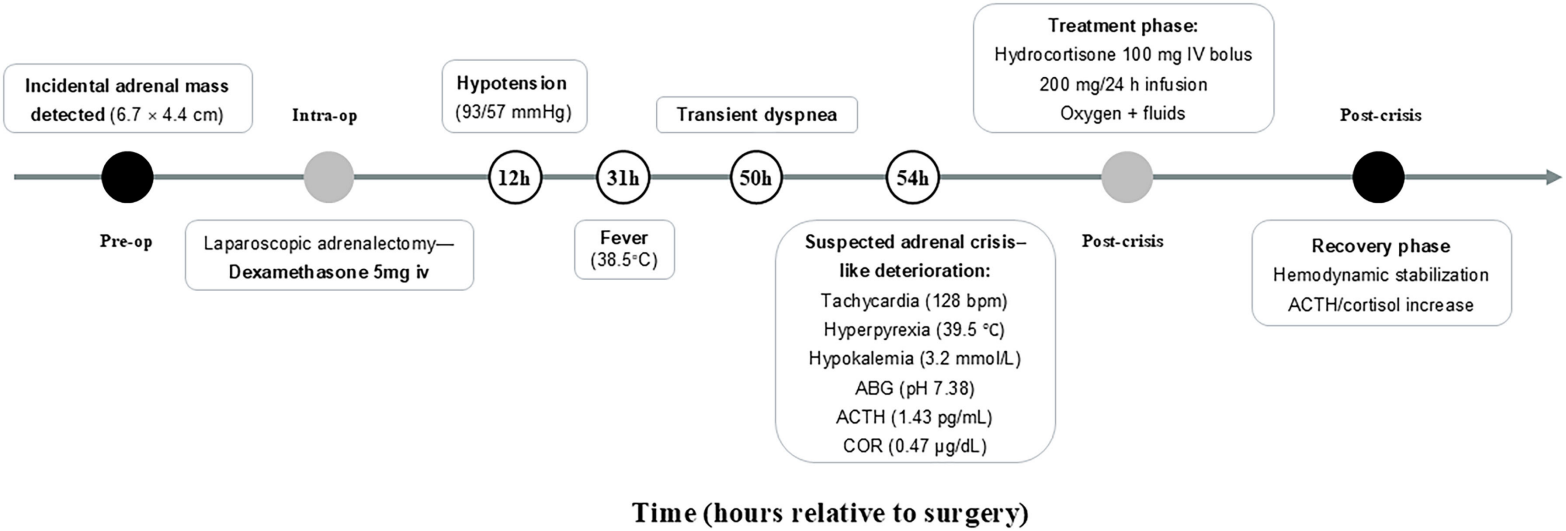
The clinical timeline of perioperative endocrine and hemodynamic changes. Pre-op, Pre-operation; Intra-op, Intra-operation; ACTH, adrenocorticotropic hormone; COR, cortisol.

The patient was discharged on oral hydrocortisone replacement and remained clinically stable. Follow-up CT at 1 month confirmed complete tumor resection without recurrence (**Supplementary Figure 2**). The clinical timeline is summarized in **Figure 3**.

### Patient perspective

The patient reported that the preoperative diagnosis of a “non-functional” tumor had been reassuring, and she expected a routine postoperative recovery. The onset of severe symptoms approximately two days after surgery was distressing for both her and her family. She expressed gratitude to the clinical team for the prompt recognition and treatment of her condition. This experience has increased her awareness of the importance of postoperative follow-up and medical adherence. She hopes that sharing her experience may help increase awareness of potential perioperative complications in similar clinical settings.

## Discussion

This case describes postoperative adrenal crisis–like deterioration following adrenalectomy for a radiologically and biochemically silent adrenal tumor in the setting of perioperative dexamethasone exposure. The primary challenge is the interpretation of postoperative endocrine findings in the context of overlapping perioperative physiological stressors and limited preoperative functional characterization.

### Perioperative dexamethasone and endocrine interpretation

Dexamethasone is a potent, long-acting glucocorticoid, seldom used for replacement due to its risk of iatrogenic Cushing’s syndrome and prolonged HPA axis suppression (11). While single-dose perioperative dexamethasone is widely used within enhanced recovery pathways and is generally safe in broad surgical populations (9, 10), it may transiently suppress ACTH secretion and complicate the interpretation of postoperative endocrine assessment.

Dexamethasone-induced adrenal suppression has been reported even after short-term exposure (12–14). In this case, suppressed ACTH and cortisol levels during deterioration are consistent with expected pharmacological effects. However, these findings cannot distinguish drug-related suppression from inter-individual variability in stress-related cortisol responsiveness, nor can they establish baseline adrenal reserve. Accordingly, dexamethasone exposure should be interpreted as a temporally associated perioperative factor rather than a causal trigger.

### Preoperative endocrine evaluation

Preoperative assessment did not include a 1 mg overnight dexamethasone suppression test; therefore, mild autonomous cortisol secretion could not be excluded according to current guideline recommendations (8). Importantly, the DST is a screening test for cortisol autonomy and does not assess adrenal stress reserve, which requires dynamic testing such as ACTH stimulation testing. Such testing was not performed due to the absence of preoperative suspicion of adrenal insufficiency, limiting full functional characterization of the hypothalamic–pituitary–adrenal (HPA) axis. In this context, basal hormonal evaluation alone is insufficient to fully capture perioperative endocrine risk. The postoperative course should therefore be interpreted considering possible occult HPA-axis vulnerability in the setting of surgical stress and perioperative dexamethasone exposure, rather than as evidence of a single causal mechanism (11).

### Diagnostic uncertainty in postoperative deterioration

The diagnosis of suspected adrenal crisis in this case is clinical and partially retrospective. Although the patient demonstrated hemodynamic instability with subsequent improvement after glucocorticoid therapy, alternative postoperative complications cannot be fully excluded. Infection, pulmonary embolism, postoperative bleeding, and systemic inflammatory responses may present with overlapping clinical and biochemical features in the early postoperative period. Because comprehensive diagnostic imaging and microbiological investigations were not completed during the acute phase, these alternatives cannot be definitively ruled out.

### Therapeutic interpretation

Clinical stabilization occurred following combined interventions including fluid resuscitation, oxygen supplementation, antipyretic therapy, and stress-dose hydrocortisone. The concurrent administration of multiple therapies precludes attribution of recovery to a single intervention. Post-treatment cortisol and ACTH levels obtained during hydrocortisone administration cannot be interpreted as indicators of endogenous HPA-axis recovery, as exogenous hydrocortisone is measured by cortisol immunoassays and suppresses ACTH secretion.

### Hypokalemia

Hypokalemia observed during deterioration is not a classical feature of adrenal crisis. Potential mechanisms include catecholamine-mediated intracellular potassium shifts, insulin/glucose-related redistribution, perioperative fluid administration, renal or gastrointestinal losses, and mixed acid–base disturbances. However, the exact mechanism could not be determined from the available data.

### Clinical implications

While a single case cannot establish practice recommendations, several clinical considerations may be relevant for perioperative management in adrenal surgery.

Guideline-recommended evaluation of cortisol autonomy, including the 1 mg overnight dexamethasone suppression test, should be performed whenever feasible in patients with adrenal incidentalomas. In selected patients undergoing adrenalectomy—particularly those with large or indeterminate tumors, borderline hormonal findings, suspected mild autonomous cortisol secretion, or concern for limited adrenal reserve—dynamic assessment of adrenal function, such as ACTH stimulation testing, may be considered.

When perioperative glucocorticoid coverage is required, shorter-acting agents such as hydrocortisone may facilitate more straightforward postoperative interpretation of endocrine status. In contrast, long-acting agents such as dexamethasone may complicate biochemical assessment due to prolonged hypothalamic–pituitary–adrenal axis suppression. However, evidence guiding optimal agent selection in adrenal surgery remains limited, and management should be individualized.

Clinicians should also be aware that postoperative adrenal crisis–like deterioration most commonly occurs within 24–72 hours after surgery. Early recognition of clinical instability, prompt measurement of cortisol and ACTH, and a low threshold for empiric hydrocortisone administration may facilitate timely diagnosis and management.

### Limitations

The inherent limitations of a single-case report apply. This episode was interpreted as suspected adrenal crisis, but it cannot be definitively confirmed, and a causal relationship with dexamethasone cannot be established. A complete exclusion of alternative postoperative complications was not possible, as evaluation for conditions such as infection, pulmonary embolism, and postoperative bleeding was not fully performed during the acute deterioration due to clinical urgency. Preoperative endocrine assessment did not include a 1 mg overnight dexamethasone suppression test, precluding exclusion of mild autonomous cortisol secretion, and dynamic testing of adrenal reserve using ACTH stimulation was not performed. In addition, post-treatment cortisol measurements obtained after hydrocortisone administration cannot be interpreted as endogenous HPA-axis recovery. The tumor was a rare ganglioneuroma, and the findings may not be generalizable to all adrenal incidentalomas.

## Conclusion

Basal endocrine evaluation alone may be insufficient to fully characterize perioperative adrenal risk. In this case, postoperative deterioration occurred in temporal association with perioperative dexamethasone exposure; however, a causal relationship cannot be established. These findings suggest that caution is warranted in interpreting adrenal tumors labeled as “non-functional” based on basal testing alone, and that perioperative endocrine context should be considered in selected patients undergoing adrenal surgery.

## Data Availability

The original contributions presented in the study are included in the article/**Supplementary Material**. Further inquiries can be directed to the corresponding authors.
