# Supplementary material for: Case report: Suspected adrenal crisis after non-functional adrenal tumor resection: a case of temporal association with perioperative dexamethasone
Source: Front Endocrinol (Lausanne). 2026 Jul 21;17:1829308. doi: 10.3389/fendo.2026.1829308 (PMC13433212; doi:10.3389/fendo.2026.1829308)
Supplement: Supplementary file 2 [file SupplementaryFile1.docx]

**Supplementary Figure 1.** Preoperative multiplanar abdominal CT images of the right adrenal mass. Coronal (A-C) and sagittal (D-F) views show a well-demarcated right adrenal lesion (arrows) across the unenhanced, arterial, and venous phases, respectively. Non-enh, AP, and VP indicate unenhanced, arterial phase, and venous phase imaging, respectively.

**Supplementary Figure 2.** Follow-up postoperative abdominal CT imaging after right adrenalectomy. (A-C) Axial CT images of the operative bed (arrows): A, unenhanced phase; B, arterial phase; C, venous phase, demonstrating complete resection without evidence of residual right adrenal mass. Non-enh, AP, and VP indicate unenhanced, arterial phase, and venous phase imaging, respectively.
