# Supplementary material for: Case report: Suspected adrenal crisis after non-functional adrenal tumor resection: a case of temporal association with perioperative dexamethasone
Source: Front Endocrinol (Lausanne). 2026 Jul 21;17:1829308. doi: 10.3389/fendo.2026.1829308 (PMC13433212; doi:10.3389/fendo.2026.1829308)
Supplement: Supplementary file 3 [file Table1.docx]

**Supplementary Table 1. Preoperative Hormonal and Biochemical Profile**

| **Parameter** | **Value** | **Reference range** | **Status** |
| --- | --- | --- | --- |
| COR (μg/dL) | 27.06 | 6.58–28.51 | N |
| ACTH (pg/mL) | 30.49 | 5.00–60.00 | N |
| K+ (mmol/L) | 4.22 | 3.50–5.50 | N |
| ALD (pg/ml) | 115.59 | 65.00–295.70 | N |
| Renin (ng/ml/h) | 5.02 | 0.93–-6.56 | N |
| ARR | 2 | <30 | N |
| DA (pmol/L) | 102.00 | ≤195.70 | N |
| E (pmol/L) | 150.46 | ≤769.00 | N |
| NE (pmol/L) | 5802.20 | 1182.80–10054.00 | N |
| 3-MT (nmol/L) | <0.08 | <0.18 | N |
| MN (nmol/L) | 0.16 | 0.08–0.51 | N |
| NMN (nmol/L) | 0.84 | 0.12–1.18 | N |

**Note:** COR, cortisol; ACTH, adrenocorticotropic hormone; K⁺, potassium; ALD, aldosterone; ARR, aldosterone-to-renin ratio; DA, dopamine; E, epinephrine; NE, norepinephrine; 3-MT, 3-Methoxytyramine; MN, netanephrine; NMN, normtanephrine; N, normal.
